# The Role of Tryptophan Metabolism in Alzheimer’s Disease

**DOI:** 10.3390/brainsci13020292

**Published:** 2023-02-09

**Authors:** Karl Savonije, Donald F. Weaver

**Affiliations:** 1 Krembil Research Institute, Toronto Western Hospital, 60 Leonard Avenue, Rm 4KD477, Toronto, ON M5T 0S8, Canada; 2 Departments of Medicine (Neurology) and Chemistry, University of Toronto, Toronto, ON M5T 0S8, Canada

**Keywords:** Alzheimer’s disease, dementia, tryptophan, autoimmune, neuroinflammation

## Abstract

The need to identify new potentially druggable biochemical mechanisms for Alzheimer’s disease (AD) is an ongoing priority. The therapeutic limitations of amyloid-based approaches are further motivating this search. Amino acid metabolism, particularly tryptophan metabolism, has the potential to emerge as a leading candidate and an alternative exploitable biomolecular target. Multiple avenues support this contention. Tryptophan (trp) and its associated metabolites are able to inhibit various enzymes participating in the biosynthesis of β-amyloid, and one metabolite, 3-hydroxyanthranilate, is able to directly inhibit neurotoxic β-amyloid oligomerization; however, whilst certain trp metabolites are neuroprotectant, other metabolites, such as quinolinic acid, are directly toxic to neurons and may themselves contribute to AD progression. Trp metabolites also have the ability to influence microglia and associated cytokines in order to modulate the neuroinflammatory and neuroimmune factors which trigger pro-inflammatory cytotoxicity in AD. Finally, trp and various metabolites, including melatonin, are regulators of sleep, with disorders of sleep being an important risk factor for the development of AD. Thus, the involvement of trp biochemistry in AD is multifactorial and offers a plethora of druggable targets in the continuing quest for AD therapeutics.

## 1. Introduction

From a global perspective, dementia is an evolving healthcare crisis, with dementia risk being strongly correlated to age and with afflicted individuals typically demonstrating decreased survival compared to age-matched cohorts [1,2,3]. Alzheimer’s disease (AD) is the most common type of dementia, accounting for more than 70% of all diagnoses. Alzheimer’s disease has classically been defined by the presence of β-amyloid (Aβ) plaques and tau neurofibrillary tangles, combined with a loss of brain volume [4,5,6]. As these markers are difficult to assess during the early stages of the disease, diagnosis has historically been suggested by a decline in cognitive function, with a definitive diagnosis being best achieved by means of post-mortem brain analysis.

Despite its immense world-wide socioeconomic impact, coupled with decades of biomedical research, there are no curative disease-modifying drugs available for AD. Therapeutic approaches have usually targeted the neurotoxic proteopathy associated with the misfolding and aggregation of Aβ and tau; however, to date, such approaches have demonstrated limited utility. Accordingly, the need to identify alternative druggable biochemical pathways is an ongoing research priority in the AD research realm. The metabolic pathways of amino acids are emerging as potentially important players in the panoply of molecular participants in AD pathology, with tryptophan metabolism being a front runner [7].

Tryptophan (trp) is an essential amino acid centrally implicated in the molecular pathogenesis of multiple neurological disorders, including major depressive disorder (MDD) and dementia [8]. Trp is metabolized to serotonin (5-hydroxytryptamine, 5-HT) via initial conversion to 5-hydroxytryptophan, catalyzed by tryptophan hydroxylase, and then to 5-HT, catalyzed by aromatic amino acid decarboxylase. 5-HT is a well-studied neurotransmitter that is the target of multiple potent neuroactive agents, ranging from therapeutic antidepressant selective serotonin reuptake inhibitors (SSRIs) to recreational serotonin-releasing agents (SRAs) such as amphetamines [9]. Beyond these obvious psychotropic indications, trp and its various metabolites may likewise be starting-point candidates in the rational design of small-molecule therapeutics for AD and related dementias for a variety of pathogenic and mechanistic reasons. Herein, multiple trp-influenced biomolecular processes implicated in AD are summarized and reviewed.

## 2. Tryptophan Metabolism

Trp is an essential amino acid which higher trophic lifeforms are incapable of producing in vivo and must acquire from dietary sources. Trp is required for protein formation, as well as being catabolized into a variety of bioactive metabolites, primarily via two distinct metabolic pathways: the serotonin pathway leading to melatonin, and the kynurenine pathway leading to the nicotinamides. Each pathway produces biologically important regulatory chemical intermediates, involved in regulating neural function, immune response, and metabolism [10].

### 2.1. Serotonergic Pathway

**Figure 1:**
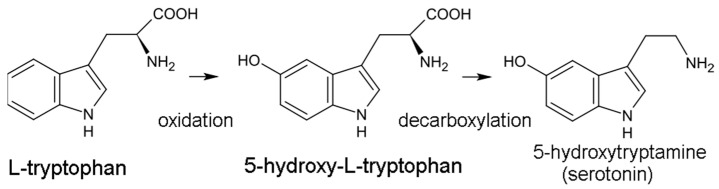
Conversion of tryptophan to serotonin.

The serotonin pathway is the less dominant of the two pathways, responsible for generating the 5-HT neurotransmitter. In this pathway, trp is initially hydroxylated to 5-HTP by tryptophan hydroxylase (TPH) then decarboxylated to 5-HT by aromatic acid decarboxylase (DDC) (Figure 1). TPH is part of a family of biopterin-dependent aromatic amino acid hydroxylase enzymes. TPH requires a reduced pteridine cofactor, molecular oxygen, and non-heme iron to hydroxylate its substrate L-tryptophan. In humans, as well as in other mammals, there are two distinct TPH genes; in humans, these genes are located on chromosomes 11 and 12 and encode two different homologous enzymes *TPH1* and *TPH2.* Since the conversion of 5-HTP to 5-HT is facile and rapid, TPH-mediated hydroxylation is the rate-limiting step in 5-HT production. 5-HT synthesis is largely confined to the central nervous system (CNS), although the gut microbiome may also be an important source of 5-HT. 5-HT is eventually converted into the hormone melatonin, involved in sleep and circadian rhythm regulation.

### 2.2. Kynurenic Pathway

**Figure 2:**
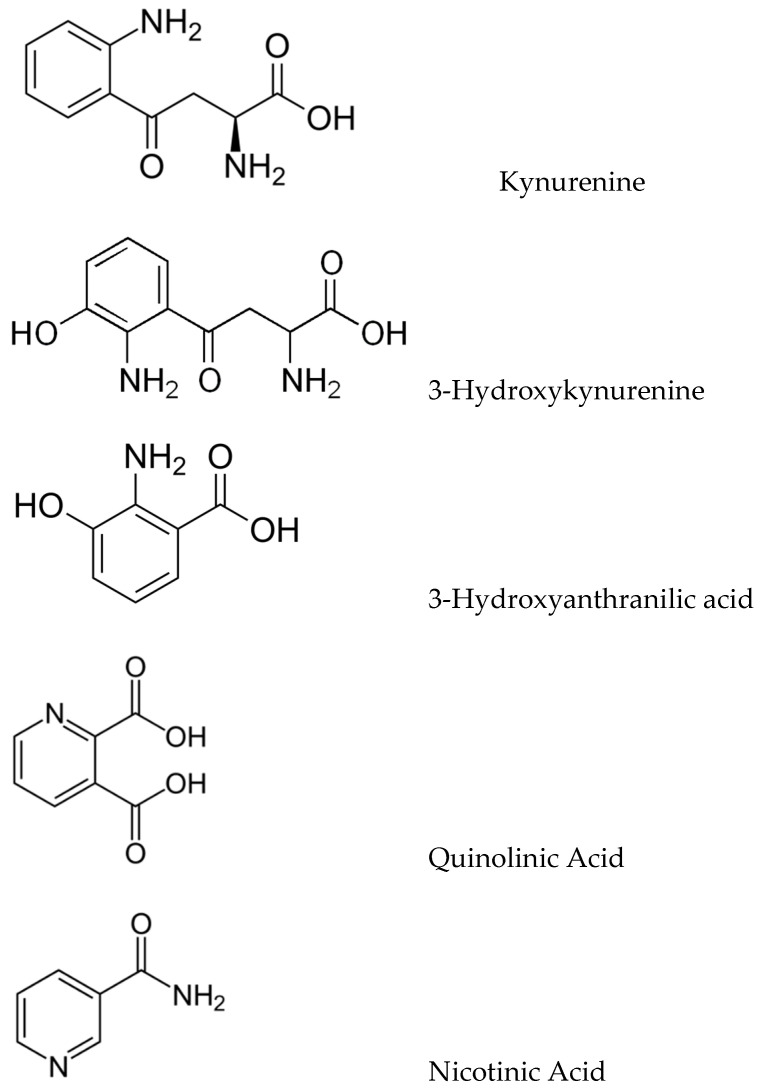
Structures of key kynurenic metabolites.

The kynurenic pathway (KP) produces a variety of biologically important kynurenic compounds and is the dominant catabolic pathway of trp metabolism. The first and rate-limiting step is the conversion of trp to *N*-formylkynurenine by either indoleamine 2,3-dioxygenase (IDO-1) or tryptophan 2,3-dioxygenase (TDO), followed by rapid *N*-formylkynurenine formamidase-catalysed conversion to kynurenine (KYN). KYN metabolism in the CNS follows two pathways: astrocytes convert KYN to kynurenic acid (KYNA) as catalyzed by kynurenine aminotransferases (KATs), whilst microglia convert KYN to 3-hydroxykynurenine (3-HK) via kynurenine monooxygenase (KMO). 3-HK is converted to 3-hydroxyanthranilic acid (3-HAA) and then to quinolinate (QUIN), which is eventually catabolized into nicotinamide (Figure 2).

Metabolites involved in the kynurenine pathway include tryptophan, kynurenine, kynurenic acid, xanthurenic acid, quinolinic acid, and 3-hydroxykynurenine. However, ultimately, this pathway leads to the production of nicotinamide adenine dinucleotide (NAD^+^), which plays a critical role in generating cellular energy. Because energy requirements are substantially increased during a neuroimmune response, the KP is a key regulator of the immune system in the brain [11]. Moreover, many kynurenines are neuroactive, modulating neuroplasticity and/or exerting neurotoxic effects in part through their effects on NMDA receptor signaling and glutamatergic neurotransmission, thus impacting emotion, cognition, pain, metabolic function, and aging, and in doing so potentially increasing the risk of developing neurodegenerative disorders [12]. In the brain, KYN is processed by either astrocytes or microglia to produce distinct neuroactive compounds that have been shown to alter not only glial-mediated immunological processes but also downstream synaptic glutamatergic neurotransmission; therefore, the KP facilitates communication between the brain and the immune system, constituting a conceptual confluence of immunologic, monoaminergic, and glutamatergic processes at the neural-immune homeostatic interface.

Via the serotonergic and kynurenic pathways, trp and trp metabolites have the capacity to influence the etiopathogenesis of AD by means of multiple mechanistic routes.

## 3. Tryptophan and Alzheimer’s Disease Pathogenesis

### 3.1. Trp and Proteopathy in AD

While the causative contributions of the ‘amyloid hypothesis’ to AD have come into question in recent years, deposits of misfolded Aβ oligomers and higher-order aggregates (e.g., fibrils), the so-called amyloid plaques, are strongly correlated with the well-described neuropathology of AD [4]. β-Amyloid aggregates (particularly small oligomers) are generally considered to be neurotoxic and neuroinflammatory, and even if not the root cause of AD, have a pronounced cytotoxic effect and possibly contribute to disease progression [13].

The biosynthesis of Aβ has received considerable study. Amyloid precursor protein (APP) is cleaved by amyloid secretase, an enzyme with several variants that cleave APP at the designated α, β and γ sites [4]. Generally, APP may be cleaved either by α-secretase, or alternatively by β-secretase then γ-secretase [4], with this latter route being responsible for the production of sAPPβ and the Aβ monomer. APP cleaving is thought to be competitive, suggesting that Aβ deposition could be downregulated by increasing the production of the α-secretase, as the α cleavage site is within the Aβ domain, and hence, sAPPα cannot produce Aβ [14]. There has been interest in α-secretase as a therapeutic target as sAPPα has potent neuroprotective actions against glutamate neurotoxicity, Aβ peptide-induced oxidative injury, and glucose deprivation. The investigation of sAPPα as a therapeutic target has indicated that trp’s serotonergic signaling pathways in the CNS are involved in the upregulation of α-secretase production; moreover, 5-HT_4_ agonists stimulate increased α-secretase production, as well as reduced amyloid burden and decreased neuroinflammation, indicating that sAPPα inhibition may have some efficacy in mitigating the pathology of AD progression [15].

In terms of other trp-influenced enzymes implicated in Aβ biochemistry, neprilysin (NEP) is a metalloproteinase regulating the brain clearance of Aβ peptides; a decrease in Aβ elimination may be a significant contributor to AD pathogenesis [16]. Two trp metabolites, 5-hydroxyindole-acetic acid (5-HIAA) and KYNA, stimulate NEP activity/expression to prevent Aβ peptide-induced neurotoxicity, possibly by interacting with the aryl hydrocarbon receptor. These data suggest promising perspectives for the design of a tryptophan metabolite-based enzyme targeting therapies against AD [17].

In addition to trp metabolism’s influence on enzymes involved in the biosynthesis of Aβ, various trp metabolites have also demonstrated the ability to directly inhibit Aβ oligomerization and aggregation. The trp metabolite 3-hydroxyanthranilic acid, generated via the kynurenic pathway, has the capacity to function as an endogenous inhibitor of Aβ aggregation [18].

Therefore, trp and trp metabolites are able to modulate Aβ biochemistry in a potentially beneficial manner by interacting with various enzymes, including α-secretase and neprilysin, and by directly interacting with Aβ itself.

### 3.2. Trp and Sleep Disorders in AD

The effects of sleep deprivation or poor quality of sleep have long been anecdotally evident, and voluminous research backs up the powerful effects of sleep on health [19,20]. In an era during which 4% of North American adults report using sleeping aids [21], the so-called sleeplessness epidemic is likely to have an ongoing negative impact on health outcomes—of concern, the sleeplessness epidemic might overlap with the AD epidemic. Recent findings have correlated disturbed sleep in middle-aged and older adults with a 30% increase in AD risk compared to those with healthy sleeping patterns [22].

Sleep has important roles in learning and memory consolidation. Not surprisingly, there are accumulating data suggesting that sleep disorders contribute to cognitive decline and the development of AD [23]. People who sleep six hours or less per night are more likely to develop Alzheimer’s dementia later in life, an observation which suggests that inadequate sleep duration increases dementia risk [24]. Common sleep-related symptoms found in AD include difficulties in falling asleep, easy arousal at night, repeated awakenings, and waking up too early in the morning with consequent sleepiness and frequent naps during the day. Amongst sleep disorders commonly occurring in people living with AD, the most frequent include sleep breathing disorders and restless legs syndrome [25]. Mechanistically, disordered sleep may influence circadian fluctuations in the concentration of Aβ in interstitial brain fluids and in glymphatic brain fluids; sleep disorders also promote the biosynthesis of Aβ.

Studies have shown Aβ levels in cerebrospinal fluid (CSF) increase throughout the day and fall away overnight, as well as a relationship between fragmented sleep and increased amyloid burden [26]. Additionally, other studies have linked sleep deprivation with increased Aβ and extracellular tau, both linked to the pathology of AD [27,28,29]. In humans, sleep deprivation has been shown to promote neuroinflammation through the increased production of pro-inflammatory cytokines including the interleukins IL-1β, IL-6 and IL-17 [30]. Inflammation has been strongly linked to the progression of AD as part of the inflammatory theory of AD, and as such, sleep deprivation appears to function as a double-edged sword, promoting negative effects through inflammation, while also impeding the potentially beneficial clearance of Aβ that happens during normal sleep. Sleep disorders are a common co-morbidity in dementia and have been suggested to promote Aβ biosynthesis and deposition [28,30].

Since trp metabolism is an endogenous regulator of the sleep cycle and since trp supplementation has been shown to aid sleep, the role of trp upon AD risk and pathogenesis via the physiological function of sleep is another important connection between trp and AD [31]. Trp has a long-recognized sedative effect, and the serotonergic metabolites 5-HT and melatonin have also been demonstrated to have a beneficial effect on sleep [22,32]. The relationship between the trp metabolite 5-HT and sleep is complex. Based on electrophysiological, neurochemical, genetic, and neuropharmacologic studies, evolving data demonstrate that 5-HT functions predominantly to promote wakefulness and to inhibit rapid eye movement sleep via the serotonergic innervation of the cerebral cortex, amygdala, basal forebrain, thalamus, preoptic and hypothalamic areas, raphe nuclei, locus coeruleus, and pontine reticular formation which comes from the dorsal raphe nucleus.

There is evidence that trp and melatonin supplements can exert sleep-related effects, increasing the quality of sleep and inhibiting Aβ formation [33]. Melatonin is a terminal metabolite in the serotonergic metabolic path and is a highly conserved molecule in all aerobic organisms. Its primary role appears to be influencing the sleep–wake cycle, as melatonin synthesis exhibits pronounced light–dark variation, with levels rising to a maximum in the early morning and decreasing throughout the day [32]. While in mammalian lifeforms, circulating melatonin is primarily produced in the pineal gland, mitochondria also produce melatonin as an antioxidant, as melatonin is an effective scavenger of oxygen radicals (O_2_^−^·) as well as the hydroxyl radical (OH·) and nitric oxide (NO·).

Since trp is prerequisite for the synthesis of melatonin, plasma trp levels exert a notable impact on the quality and duration of sleep. Trp supplementation or diets which promote an increase in trp uptake have a demonstrated positive effect on sleep onset, duration, and quality [34]. This connection with sleep neurochemistry may be part of the complex relationship between trp and AD.

### 3.3. Trp and Kynurenic Neurotoxicity in AD

Tryptophan conversion to kynurenine accounts for more than 95% of all trp catabolism; the first and rate-limiting step in this pathway is the conversion of trp to KYN by the enzymes IDO and TDO [35]. TDO is primarily produced in the liver and is responsible for the majority of KYN production [35], while IDO exists in two subtypes, IDO-1 and IDO-2. IDO-1 is produced in many different tissues as an anti-inflammatory immune-downregulating signal pathway; IDO-2 is less common [36]. KYN can then be converted to KYNA, a neuroprotective compound, which is subsequently metabolized into 3-hydroxyanthranilic acid (3-HAA) via anthranilic acid (AA) or 3-hydroxy-L-kynurenine (3-HK). 3-HAA then breaks down into either picolinic acid (PIC) or quinolinic acid (QUIN), the latter of which is the precursor to NAD+. Many of these breakdown products are neuroactive: AA, 3-HAA and KYNA are typically considered to be neuroprotective, whilst 3-HK and QUIN are typically considered neurotoxic [37]. This makes the kynurenic pathway (KP) of particular interest to the study of neurodegenerative diseases such as AD.

QUIN is one of the principal neurotoxic metabolites of trp. QUIN is able to complex with Fe(II), inducing the formation of the hydroxyl radical (•OH), and other reactive oxidant species (ROS), indicating that QUIN may be a potent mediator of oxidative stress (OS). Furthermore, QUIN-mediated OS and tau hyperphosphorylation are strongly linked, synergistically augmenting the neurotoxicities of both chemical entities [38]. Neurofibrillary tau tangles (NFTs) are a result of tau hyperphosphorylation and significantly contribute to the neurotoxic proteopathy of AD; NFTs contribute to neuronal death and the loss of cognitive ability [5]. QUIN, the KP metabolite downstream of the kynurenine monooxygenase (KMO) enzyme, co-localizes with NFTs, indicating that KMO inhibition may have a complex therapeutic effect against AD by promoting the production of the neuroprotective KP metabolites KYNA and 3-HAA while simultaneously reducing the production of QUIN and potentially impacting the formation of NFTs [39]. The goal of reducing QUIN neurotoxicity has thus led to the consideration of the trp metabolism KMO enzyme as a therapeutic target for AD [40].

The inhibition of both the IDO-1 and KMO enzymes has become a subject of interest in recent years due to their potential implications in neurodegenerative diseases such as AD. IDO-1 inhibitor research is already well developed in the field of oncology, with IDO-1 playing a potential role in tumor immune escape. IDO-1 inhibitors have demonstrated the amelioration of AD symptoms in rodent trials [41]. KMO inhibition has also been shown to strongly and beneficially drive the increased production of KYNA in vitro, as well as in rodent models [42]. Unfortunately, the penetration of the blood–brain barrier (BBB) has become a significant stumbling block for IDO inhibition, and while some tools exist for the rational design of brain-penetrant IDO-1 inhibitors [43], no drug candidates have emerged to date. Likewise, KMO inhibitors must also contend with the BBB, as well as potential unfavorable interactions with the FAD cycle leading to significantly increased hydrogen peroxide generation [44].

Fully understanding the role of the KP in neurodegenerative disease is a subject of much recent interest as it appears to exhibit both neurodegenerative and neuroprotective properties. Generally, it has been accepted that an excess of KYN will lead to the increased production of neurotoxic intermediates, and as such, research into therapeutic targeting of the KP has largely focused on the inhibition of key enzymes such as IDO-1 [45]. IDO-1 is an inflammation-upregulated enzyme that catalyzes the conversion of trp to KYN, the rate-limiting step in the KP, and as such, it is the obvious target for any KP-based interventions. However, despite its clear therapeutic potential, IDO-1 inhibition has yet to progress past murine models. A new target of interest may be KMO, the enzyme responsible for the metabolism of KYN to 3-HK. KMO inhibition could preferentially shift the KP away from neurotoxic metabolites 3-HK and QUIN and instead toward the neuroprotective KYNA metabolite, providing a therapeutic effect on the reduction in inflammatory action due to the KP [46].

### 3.4. Trp and Neuroinflammation in AD

Among the predominant sources of KMO in the CNS are microglia, the first cellular line of response for the CNS immune system. In the classic understanding of microglia, resting-state microglial cells are quiescent and dendritic, extending sensory probes into the brain’s microenvironments and monitoring for stimuli; once activated, they shift into a mobile amoebic form, dubbed “activated microglia” and start expressing pro-inflammatory cytokines such as TNF-α, IL-6, IL-1β, and IFN-γ, as well as chemokines to signal other microglia [47]. As technology has evolved, a deeper understanding of activated microglia has developed, with the identification of several ‘alternative’ activation states. ‘Alternatively’ activated microglia were dubbed M2-activated, while ‘classically’ activated microglia were renamed to M1-activated [48]. M2 were then subdivided further based on specific cytokine production, as well as chemokine secretion and function, with a generally accepted M2a, M2b, and M2c subtype [49,50]. Improved in vivo analysis then began to show microglia demonstrating hybrid phenotypes and suggested microglial activation is instead a spectrum, with microglia able to tailor phenotypes as needed based on the local environment. Generally, though, the M1-M2 axis is useful, though dated, shorthand for differentiating between the more pro-inflammatory and the more anti-inflammatory states of microglial activation, and it will be the terminology used going forward in this review.

In the case of a typical inflammatory episode, such as an invading pathogen or tissue damage, pro-inflammatory cytokines, especially INF-γ and TNF-α, signal the microglia to assume an M1-activated state, whereby they deploy a wide range of immune receptors including Toll-like receptors (TLRs), nucleotide-binding oligomerization domains (NODs), NOD-like receptors, and scavenger receptors [51]. These M1-type microglia recruit more M1-type microglia via the production of M1-polarizing cytokines, present antigens and express inducible NO as part of an effort to neutralize invading pathogens. While a necessary part of the immune response, M1-type microglia create high levels of local oxidative stress, and if not downregulated, can perpetuate the recruitment of additional M1-type microglia through the continued production of M1-polarizing cytokines, potentially creating a vicious cycle of inflammation begetting further inflammation—a cytotoxic hypercytokinemic state [52]. The anti-inflammatory M2 phenotypes are predominately triggered by the IL-4 cytokine IL-4; however, a wide range of different M2-type phenotypes can be triggered by various anti-inflammatory cytokines, including IL-3, IL-10, IL13, glucocorticoids, TLRs and other receptors, demonstrating the impressive plasticity of microglial phenotypes [53,54]. In contrast to the M1-type activated state, M2-type microglia are more involved in restoring homeostasis and assisting healing by secreting anti-inflammatory and neurotrophic factors [54].

While M1-type activation is usually a response to an acute event, chronic low-level inflammation can create a ‘priming’ effect, in which microglia are more easily activated and the overall phenotyping skews towards M1 [55]. As part of their function, M1 microglia generate ROS and reactive nitrogen species, which increases stress on the CNS, and sustained M1 activation has been implicated in the pathogenesis of multiple neurodegenerative disorders. This ‘priming’ effect may explain the overexpression of M1 in several neurodegenerative disorders, notably including AD, in which M1-type microglia are observed in high concentrations in the local region surrounding NFTs and amyloid plaques [56]. The clearance of Aβ is a function of activated microglia; however, M1-activated microglia demonstrate impaired Aβ and perpetuate the chronic inflammation local to the plaques via the production of pro-inflammatory cytokines [57]. As a result, inactivation or phenotype transition from M1 towards M2 has become a subject of recent interest in the fight against AD, potentially able to provide a double effect of reducing amyloid burden while also reducing the inflammatory factors that have been proposed to contribute to the progression of the disease.

Given the potential importance of M1/M2 polarity distribution to AD pathogenesis, factors which influence this ratio emerge as relevant neurochemical compounds. Trp metabolites have the capacity to fulfill this role; for example, the 3-hydroxyanthranilate metabolite has the ability to reduce IL-6 and TNF-α inflammatory cytokine production. Conversely, it has been reported that IL-1β and IL-6 potentiate the metabolic pathways from trp to KA and QUIN, suggesting that M1 microglia can decrease 5-HT production. Therefore, neuroinflammation represents yet another biomolecular mechanism whereby trp and trp metabolites may exert a regulatory influence on AD progression.

### 3.5. Trp and Innate Autoimmunity in AD

Neuroinflammation is a product of the neuroimmune system, and not surprisingly, the disordered function of brain immunity is another postulated disease mechanism for AD; in this mechanistic conceptualization, AD is a fundamental chronic, progressive disease of the neuroimmune system. Within the spectrum of possible immunotoxicities contributing to AD, the concept of AD as an autoimmune disease has recently gained attention [58,59,60]. In this concept of AD as an autoimmune disease model, the following sequence of events is suggested to cause AD: in response to either pathogen-associated molecular pattern-stimulating events (e.g., infection or air pollution), or damage-associated molecular pattern-stimulating events (e.g., trauma or ischemia), Aβ is physiologically produced and released (in monomeric and multiple oligomeric aggregate forms) as an early immunopeptide responder which instigates an innate immunity cascade. Once released, Aβ, exhibiting cytokine-like immunopeptidic properties, functions in both immunomodulatory and antimicrobial roles, regardless of the stimulus that prompted its release (i.e., infection vs. depression); thus, released Aβ is going to exhibit both immunomodulatory and “antimicrobial” properties even if no microbes are present.

The antimicrobial properties of Aβ arise from Aβ’s coulombic interactions, mediated by the cationic HHQK motif within Aβ, with anionic macromolecules in the membranes of electrophysiologically active cells, such as bacteria [59]. Regrettably, Aβ’s antimicrobial actions result in a misdirected attack upon “self” neurons, arising from the electrophysiological similarities between neurons and bacteria in terms of transmembrane potential gradients (−80 mV) and topologically similar anionic charge distributions on outer leaflet membrane macromolecules (gangliosides in neurons; cardiolipin or lipopolysaccharides in bacteria). This inadvertent, but specific, attack upon neurons, which have been mistaken for bacteria, causes membrane rupture and neuronal death by necrosis—this renders the process as autoimmune. Thus, Aβ’s antimicrobial activities enable autoimmune neurotoxicity, ultimately contributing to neuronal death via necrosis. In addition to such innate autoimmune neurotoxicity, the antimicrobial properties of Aβ also contribute to mitochondriopathy (mitochondria are bacterial endosymbionts and susceptible to antimicrobial peptides). The subsequent breakdown products of necrotic (but not apoptotic) neurons include the release of GM1-Aβ molecular complexes which diffuse to nearby neurons eliciting the further release of Aβ, thus leading to a chronic, self-perpetuating ongoing autoimmune cycle with microglial activation, pro-inflammatory cytokine release, tau aggregation, and synaptotoxicity.

Considering AD as an autoimmune disorder raises the possibility of identifying endogenous factors to ameliorate disease progression. Recently, to identify such a factor, Meier-Stephenson et al. sought to identify a compound endogenous to the human brain which could be either administered directly or used as a starting point in the design of an orally active, brain-penetrant drug [60]. They screened a library of 1137 human brain molecules (molecular weight < 650 g/mol), first using biotinylated Aβ_1–42_ anti-oligomerization and kinetic thioflavin T (ThT) Aβ_1–40_ anti-aggregation assays, and secondly using cytokine-based anti-inflammatory assays. The initial screening identified multiple ‘hits’ against Aβ aggregation within the tryptophan metabolic pathway, including both indole-based and anthranilate-based metabolites. Although trp was inactive, metabolites from its principal catabolic pathways including tryptamine, 5-hydroxytryptamine (5-HT), 5-methoxytryptamine, and 3-hydroxyanthranilate inhibited Aβ_1–42_ oligomerization and Aβ_1–40_ aggregation. In secondary anti-immunopathy screens, these same metabolites modified the release of pro-inflammatory cytokines: 5-HT, for example, modulated IL-1β production and release from microglia. Their high-throughput screening campaign suggests tryptophan metabolites as a single molecular platform for the design of putatively therapeutic agents targeting both of AD’s immunopathic–proteopathic pathologies.

Although this autoimmune mechanism is not one of the current frontrunner hypotheses for AD, this work is interesting, particularly within the context of understanding a fundamental role for trp in AD. In their high-throughput screening of all small brain molecules, trp metabolites emerged as the highest hits for endogenous compounds able to modulate both the proteopathy and immunopathy of AD.

## 4. Conclusions

The role of trp in AD is complex and controversial. Could AD arise from an imbalance between neuroprotective and neurotoxic effects arising from trp and trp metabolites? In addition, do neurotoxic metabolites from trp metabolism also possibly contribute to the neuropathology of AD? These issues await further study, but arguably, the careful manipulation of trp metabolism may afford therapeutic opportunities via multiple mechanisms.

Tryptophan metabolism plays a major role in CNS biochemistry and, unsurprisingly, has implications for the onset and progression of AD, humankind’s principal neurodegenerative disease. The kynurenic side of trp metabolism has been of particular interest as inflammatory theories of brain disease have become more prominent in the scientific discourse and synergize with new understandings of the role of microglia in the damaged brain. However, there are multiple biomolecular routes by which trp and its metabolites may contribute to AD progression. Tryptophan and its associated metabolites are able to inhibit various enzymes participating in the biosynthesis of β-amyloid, and one metabolite, 3-hydroxyanthranilate, is able to directly inhibit β-amyloid oligomerization. While certain tryptophan metabolites are neuroprotectant, other metabolites, such as quinolinic acid, are neurotoxic and may contribute to AD progression. Trp metabolites also have the ability to influence microglia and cytokines in order to modulate the neuroinflammatory and neuroimmune factors which trigger pro-inflammatory cytotoxicity in AD. Finally, trp and various metabolites, including melatonin, are regulators of sleep, with disorders of sleep being an important risk factor for the development of AD. Thus, the involvement of trp biochemistry in AD is multifactorial and offers a plethora of druggable targets in the continuing quest for AD therapeutics.

The concept that trp and its metabolites, particularly 5-HT, are involved either directly or indirectly in AD is not new. A simple PubMed search of “tryptophan” and “Alzheimer” yields 545 hits, with 305 in the past decade; similarly, a search of “tryptophan” and “serotonin” yields 1831 hits with 231 from clinical protocols and clinical trials. The aim of this review was not a comprehensive recitation of trp and conventional thinking of AD, but rather to uniquely position trp as a central player in many of the neurochemical processes of AD, and not merely as a supporting actor to the Aβ story. Trp and trp metabolism are key players in the etiopathogenesis of AD and worthy holders of the position as druggable targets.

### Future Directions

Trp metabolism is a complex biochemical process worthy of further evaluation as a source of potentially druggable targets. Inhibitors of the various enzymes (IDO and KMO) participating in trp metabolism as well as analogs of the various trp metabolites (3-hydroxyanthranilate) represent possible drug-development opportunities. Such compounds could be either de novo designed new chemical entities or potentially repurposed drugs. Since some of these targets are already being considered for other indications (e.g., IDO inhibitors for cancer), drug design lessons from these other disorders may be imported into the task of AD drug development. Finally, trp-related druggable targets may enable small-molecule therapeutics to be developed rather than biologics. Small-molecule therapeutics afford the opportunity of having the capacity to be ‘dirty drugs’ (i.e., targeting more than one receptor at once), conceivably providing a drug with the ability to concomitantly target both Aβ and neuroinflammatory mechanisms, which is different from the tightly receptor-focused mechanism of action of biologics. Small-molecule therapeutics additionally have the potential to be cost-effective and more readily produced and distributed on a global scale. AD is unquestionably a disorder of worldwide prevalence, and global diseases deserve global solutions—the therapeutic manipulation of trp metabolism may be such a solution.
